# Menopause Timing and Later-Life Cognition: A Comparative Study of China, India, and the United States

**DOI:** 10.1093/geroni/igaf122.510

**Published:** 2025-12-31

**Authors:** Muqi Guo, Tsai-Chin Cho, Alden Gross, Lindsay Kobayashi

**Affiliations:** University of Michigan School of Public Health, Ann Arbor, Michigan, United States; University of Michigan School of Public Health, Ann Arbor, Michigan, United States; Johns Hopkins Bloomberg School of Public Health, Baltimore, Maryland, United States; University of Michigan School of Public Health, Ann Arbor, Michigan, United States

## Abstract

Women have a higher dementia prevalence than men, but midlife risk factors alone do not fully explain this disparity. Estrogen, which has neuroprotective effects, declines sharply at menopause, potentially influencing cognitive function. This study examines the association between menopause age and later-life cognition in China, India, and the U.S., where cross-national comparisons are lacking. We analyzed nationally representative data from postmenopausal women aged 45+ in the China Health and Retirement Longitudinal Study (2018, n = 7,412), the Longitudinal Aging Study in India (2017–2019, n = 22,648), and the U.S. Health and Retirement Study (2018, n = 5,577). Menopause age was categorized as < 40, 40–44, 45–49, 50–55 (reference), and >55 years. Cognitive function was assessed as total score of harmonized immediate and delayed word recall. Linear regression models adjusted for socio-demographic factors, and interaction terms tested country-specific differences. Premature (< 40) and early menopause (40–44) were more common in India, while menopause at 50–55 was most frequent in the U.S. Across countries, menopause before 50 was linked to poorer cognition. In China, late menopause (>55) was also associated with cognitive decline. U.S. women with menopause at 45–49 showed a greater decline compared to their counterparts in China and India. Overall, earlier menopause is associated with lower cognitive function across all three countries, with Indian women facing a greater burden due to higher early menopause prevalence. Additionally, late menopause may pose cognitive risks for Chinese women. These findings highlight menopause timing as a key factor in cognitive aging across diverse economic contexts.

